# Hierarchical Flaky Spinel Structure with Al and Mn Co-Doping Towards Preferable Oxygen Evolution Performance

**DOI:** 10.3390/ma18153633

**Published:** 2025-08-01

**Authors:** Hengfen Shen, Hao Du, Peng Li, Mei Wang

**Affiliations:** 1 School of Materials Science and Engineering, North University of China, Taiyuan 030051, China; 15208623805@163.com; 2 School of Energy and Power Engineering, North University of China, Taiyuan 030051, China; 18649390512@163.com (H.D.); lipeng.epe@nuc.edu.cn (P.L.)

**Keywords:** spinel, bimetallic doping, hierarchical structure, dealloying, oxygen evolution reaction

## Abstract

As an efficient clean energy technology, water electrolysis for hydrogen production has its efficiency limited by the sluggish oxygen evolution reaction (OER) kinetics, which drives the demand for the development of high-performance anode OER catalysts. This work constructs bimetallic (Al, Mn) co-doped nanoporous spinel CoFe_2_O_4_ (np-CFO) with a tunable structure and composition as an OER catalyst through a simple two-step dealloying strategy. The as-formed np-CFO (Al and Mn) features a hierarchical flaky configuration; that is, there are a large number of fine nanosheets attached to the surface of a regular micron-sized flake, which not only increases the number of active sites but also enhances mass transport efficiency. Consequently, the optimized catalyst exhibits a low OER overpotential of only 320 mV at a current density of 10 mA cm^−2^, a minimal Tafel slope of 45.09 mV dec^−1^, and exceptional durability. Even under industrial conditions (6 M KOH, 60 °C), it only needs 1.83 V to achieve a current density of 500 mA cm^−2^ and can maintain good stability for approximately 100 h at this high current density. Theoretical simulations indicate that Al and Mn co-doping could indeed optimize the electronic structure of CFO and thus decrease the energy barrier of OER to 1.35 eV. This work offers a practical approach towards synthesizing efficient and stable OER catalysts.

## 1. Introduction

Society is now confronted by resource depletion and pollution escalation from overexploitation, necessitating an urgent clean energy transition for sustainable development [1,2]. With its zero carbon emissions, high energy density, and high heat of combustion, hydrogen serves as an excellent alternative carrier fuel to traditional fossil fuels [2,3]. Electrocatalytic water splitting represents the most viable and efficient route to a green hydrogen economy [4]. Since OER entails a more intricate four-electron transfer (4OH^−^ → O_2_ + 2H_2_O + 4e^−^), the development of efficient OER electrocatalysts is essential for indirectly boosting the overall efficiency [5,6]. Although noble metal catalysts such as IrO_2_ and RuO_2_ are regarded as advanced OER electrocatalysts due to their high activity and stability, their practical application is limited by resource scarcity and high costs, hindering large-scale commercialization [7]. Consequently, it is crucial to develop cost-efficient OER catalysts capable of rivaling the performance of benchmark catalysts.

Spinel oxides constitute high-performance OER electrocatalysts, synergizing excellent electrical transport properties with crystallographic tunability across tetrahedral and octahedral sublattices [8]. Through bimetallic doping techniques, atomic-level tuning of the lattice structure and electronic structure can be achieved, thereby tailoring their intrinsic activity characteristics [9,10]. For instance, Li’s team successfully developed a high-performance NdNi-Co_3_O_4_ spinel catalyst. Their work demonstrated that incorporating a second transition metal with size and charge similar to cobalt into the Co_3_O_4_ lattice enhances its catalytic activity. Furthermore, this partial doping strategy simultaneously facilitated the generation of oxygen vacancies (Vo) and improved corrosion resistance. This catalyst exhibited an ultralow overpotential of 269 mV to achieve a current density of 10 mA cm^−2^ for the OER [11]. Chen’s team restructured the Co_3_O_4_ lattice through high-temperature doping of Fe and Cr, which induced oxygen vacancy formation, enhanced electronic properties, and accelerated electron transfer during the OER [12]. These results demonstrate that by controlling the selection and concentration of dopants, it is possible to not only optimize the microstructural morphology of the spinel oxides but also regulate their electronic properties, leading to an expanded electrochemically active surface area. However, due to their high synthesis costs and complicated processes that hinder scale-up, few of these materials meet the requirements for practical applications.

Dealloying represents an elegant top-down strategy that employs selective etching of electrochemically active elements from precursor alloys, enabling spontaneous surface diffusion-mediated self-organization of inert components into bicontinuous nanoporous architectures [13,14,15]. Notably, in the dealloying process, the substantially higher etching rate relative to the rates of migration and rearrangement governs the formation of the three-dimensional nanoporous structure [16]. Wang’s team reported a facile synthesis strategy (alloying–dealloying–annealing) for preparing Mn-doped bicontinuous nanoporous CuCo_2_O_4_ spinel oxides with dual cationic and anionic defects. This material achieved a current density of 10 mA cm^−2^ at only 310 mV and exhibited a low Tafel slope of 91 mV dec^−1^ [17]. The dealloying strategy spontaneously forms bicontinuous nanoporous materials with both structural and lattice defects through selective dissolution of reactive elements from the alloy precursor. Concurrently, the undissolved active components undergo in situ doping into the host material, optimizing its electronic structure [18].

This work presents a facile two-step dealloying strategy for synthesizing bimetallic-doped spinel oxides. Initial dealloying of the Al_80_Mn_5_Co_5_Fe_10_ precursor selectively leached Al, forming a flake-like structure. Subsequent Mn removal via secondary dealloying constructs a hierarchical flake-like architecture. The bimetallic (Al and Mn) co-doped CoFe_2_O_4_ not only optimizes electronic structure but also establishes efficient mass transport channels for OER, delivering outstanding catalytic efficiency. In 1 M KOH, np-CFO (Al and Mn) achieves an OER overpotential of 320 mV at 10 mA cm^−2^. Furthermore, it demonstrates a minimal Tafel slope of 45.09 mV dec^−1^, outperforming the benchmark RuO_2_ catalyst. Long-term stability testing at 100 mA cm^−2^ demonstrates stable operation in 1 M KOH for 100 h with negligible overpotential increase. Even under industrial operating conditions (6 M KOH, 60 °C), the electrode requires only 1.83 V to sustain a current density of 500 mA cm^−2^, maintaining stable oxygen production rates for over 100 h with minimal voltage drop. Theoretical simulations indicate that Al and Mn co-doping could indeed optimize the electronic structure of CFO and thus decrease the energy barrier of OER to 1.35 eV.

## 2. Materials and Methods

### 2.1. Material Preparation

Inspired by previous reports [17,19], the initial alloy ingots (i.e., Al_85_Co_5_Fe_10_, Mn_70_Co_10_Fe_20_, Al_80_Mn_5_Co_5_Fe_10_) are put in a furnace. To enable scalable production, master alloys are processed into ribbons via melt spinning with single-roller quenching apparatus. Selective dissolution of Al and Mn is subsequently performed in 6 M NaOH (NaOH, ≥96%, Aladdin Industrial, Shanghai, China) and 1 M (NH_4_)_2_SO_4_ ((NH_4_)_2_SO_4_, ≥99%, Aladdin Industrial, Shanghai, China) solutions, respectively. Specifically, the np-CFO (Al and Mn) sample is prepared as follows: The original Al_80_Mn_5_Co_5_Fe_10_ alloy ingot is first dealloyed in a 6 M NaOH solution to remove Al. The resulting sample is washed, dried, and then subjected to a secondary dealloying step in 1 M (NH_4_)_2_SO_4_ solution to remove Mn. Finally, the sample is rinsed with deionized water and ethanol, followed by vacuum drying at 60 °C for 5 h.

### 2.2. Physicochemical Characterization

Crystal structure characterization was performed using X-ray diffraction (DX-2700BH, λ = 0.154 nm) with the X-ray tube operating at 40 kV and 30 mA. Scanning electron microscopy (SEM, JSM-7900F, JEOL, Tokyo, Japan) equipped with integrated energy-dispersive spectroscopy (EDS) was used to simultaneously capture microstructural images and perform elemental mapping analysis, directly visualizing the spatial distribution of elements within the sample. High-resolution transmission electron microscopy (TEM) imaging revealed the intricate microstructural details of the materials. The chemical states of constituent elements were analyzed via X-ray photoelectron spectroscopy to elucidate their electronic characteristics.

### 2.3. Electrochemical Measurements

The OER performance was evaluated using a CS2350H electrochemical workstation (CS2350H, CorrTest, Wuhan, China) within a standard three-electrode configuration, with Pt foil as the counter electrode, Hg/HgO as the reference electrode, and the in-house-prepared catalyst as the working electrode. To fabricate the working electrode, 4 mg of finely ground catalyst was combined with 1 mg of carbon black (Vulcan XC−72, Cabot, Boston, MA, USA) in a solution mixture containing 400 μL of isopropanol and 100 μL of 0.5 wt% Nafion (5 wt%, DuPont, Wilmington, DE, USA), followed by sonication to form a homogeneous catalyst ink. Subsequently, 10 μL of the catalyst ink was uniformly coated onto a polished glassy carbon electrode (mass loading: 0.51 mg cm^−2^) and dried under ambient conditions. For electrochemical testing, all potentials were iR-compensated for by 95% and were normalized to the reversible hydrogen electrode (RHE) using the following equation:

(1) $$E_{RHE}=E_{Hg/HgO}+0.059\times pH+0.098$$

The overpotential (η) for the OER was calculated using the following equation:

(2) $$ƞ=E_{RHE}-1.23$$

The Tafel slope was calculated from linear sweep voltammetry (LSV) polarization curves using the following equation:

(3) ƞ=b×log|j|+a

where η is the overpotential after an iR drop, b is the Tafel slope, j is the current density, and a is the Tafel constant.

To determine the electrochemical surface area (ECSA), cyclic voltammetry (CV) was first performed in a non-faradaic region (1.0–1.1 V vs. RHE) at various scan rates (5–100 mV s^−1^), where no faradaic current was observed.

By plotting Δj/2 at 1.05 V (Δj = janode − jcathode) against the scan rates, the C_dl_ value was calculated as half of the slope. Afterward, the ECSA value could be approximately evaluated from C_dl_ using the following formula:

(4) $$ECSA=\frac{C_{dl}}{C_{s}}=\frac{C_{dl}}{40\mu^{-2}{per}_{ECSA}^{2}}$$

Here, C_s_ is usually chosen as 40 µF cm^−2^.

Carbon fiber paper (CFP)-supported electrodes are essential for stability and durability testing to prevent powder catalyst detachment under prolonged bubble evolution conditions. A homogeneous slurry was prepared by uniformly dispersing a powdered catalyst, carbon black, and polyvinylidene fluoride (PVDF) in N-methyl-2-pyrrolidone (NMP) at a 70:15:15 mass ratio.

A gas-tight electrolytic system was assembled in an H-cell using symmetrically arranged np-CFO (Al and Mn) || Pt/C electrodes. Hydrogen and oxygen evolution was monitored via water displacement, with photographic documentation of the gas volumes in both the cathode and anode compartments at 10 min intervals.

### 2.4. Computational Methods

The density functional theory (DFT) framework, implemented via the Vienna Ab initio Simulation Package (VASP), underpins all computational analyses in this study. Within the DFT formalism, exchange–correlation effects were approximated using the Perdew–Burke–Ernzerhof (PBE) functional, with electron–ion interactions treated by projector augmented-wave (PAW) pseudopotentials. The DFT + U method was employed to correct standard DFT limitations for highly localized, strongly correlated systems. Hubbard U parameters of 3.4 eV (Co), 3.3 eV (Fe), and 3.1 eV (Mn) were applied. Electrostatic interactions between periodic slabs were minimized using a 15 Å vacuum layer perpendicular to the catalytic surface plane. Systematic parameter optimization yielded a 400 eV plane-wave cutoff, with ionic relaxation convergence at −0.03 eV Å-1 and an electronic SCF tolerance of 1 × 10^−5^ eV. Brillouin zone sampling was carried out with 2 × 2 × 1 Monkhorst–Pack grids, optimized for slab surface calculations.

Subsequently, the Gibbs free energy change (ΔG) for the four elementary reaction steps was determined through the following thermodynamic relationships:

(5) $${∆G}_{1}=G(*\;OH)-G(*)-G({OH}^{-})$$

(6) $${∆G}_{2}=G\left(*\;O\right)+G(H_{2}O)-G(*\;OH)-G({OH}^{-})$$

(7) $${∆G}_{3}=G\left(*\;OOH\right)-G(*\;O)-G({OH}^{-})$$

(8) $${∆G}_{4}=1.6-{∆G}_{1}-{∆G}_{2}-{∆G}_{3}$$

Here, ∗, ∗OH, ∗O, and ∗OOH represent the clean catalyst, adsorbed OH, adsorbed O, and adsorbed OOH, respectively.

## 3. Results and Discussion

### 3.1. Microstructure Analysis

**Scheme 1:**
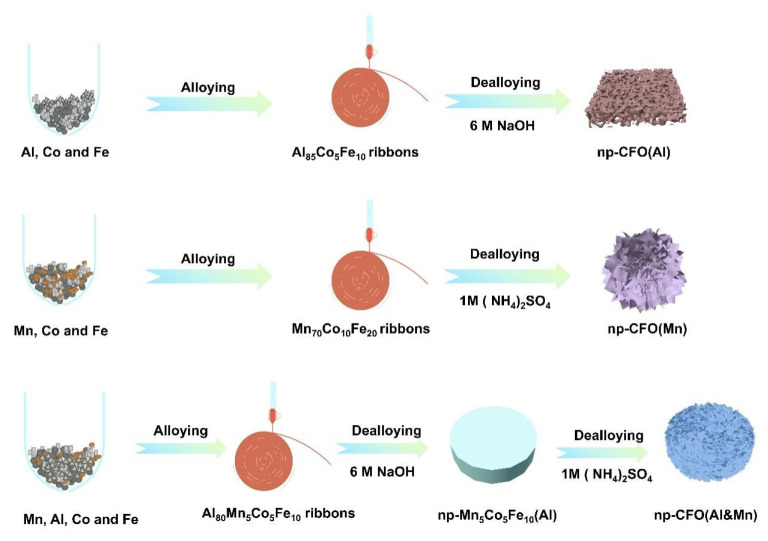
Synthesis process of np-CFO (Al), np-CFO (Mn), and np-CFO (Al and Mn).

The precursor alloys Al_85_Co_5_Fe_10_, Mn_70_Co_10_Fe_20_, and Al_80_Mn_5_Co_5_Fe_10_ were initially selected based on their optimal compositions for dealloying processes. XRD analysis (Figure S1) confirmed that Al_85_Co_5_Fe_10_ consists of Al_5_Fe_2_ (PDF#47-1435) and Fe (PDF#99-0005) phases, Mn_70_Co_10_Fe_20_ predominantly comprises FeMn_4_ (PDF#03-1180), and Al_80_Mn_5_Co_5_Fe_10_ forms the Al_13_Co_4_ (PDF#50-0694) phase. These results demonstrate excellent compositional homogeneity and high crystallinity in all precursors. Subsequent partial dealloying (Scheme 1) yields three nanoporous electrodes, including Al-doped, Mn-doped, and Al-Mn co-doped CoFe_2_O_4_. Phase analysis confirms that np-CFO (Al), np-CFO (Mn), and np-CFO (Al and Mn) all consist of the CoFe_2_O_4_ phase (PDF#01-079-1744, Figure 1a). Among these, np-CFO (Al and Mn) exhibits better crystallinity than the other two. Furthermore, an obvious negative shift in peaks in np-CFO (Al and Mn) can be observed compared to the standard diffraction peaks. This shift signifies the significant incorporation of Al and Mn atoms into the lattice [20]. This interpretation aligns with Vegard’s law and Bragg’s law, which relate peak position to interplanar spacing.

**Figure 1:**
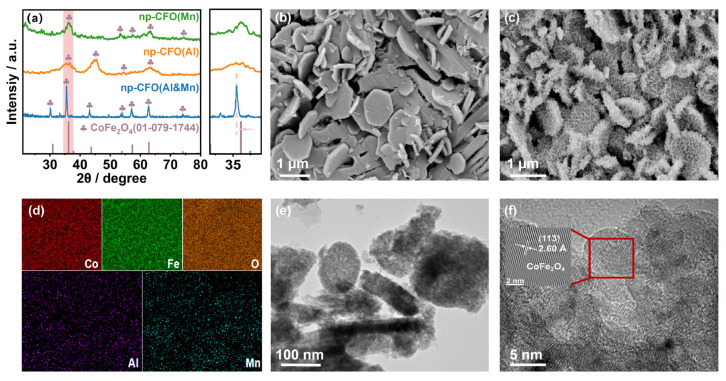
(**a**) XRD patterns of np-CFO (Al), np-CFO (Mn), and np-CFO (Al and Mn). The pink dashed lines represent the diffraction peaks of np-CFO (Al and Mn), with the inset showing a noticeable negative shift in its diffraction peaks compared to the standard peaks; (**b**,**c**) SEM images of np-CFO (Al and Mn) through initial and secondary dealloying; (**d**) corresponding elemental mappings; (**e**,**f**) TEM and HRTEM of np-CFO (Al and Mn).

Figure 1b,c show SEM images of the morphology of np-CFO (Al and Mn) obtained from the initial and secondary dealloying steps. The initial dealloying treatment of the Al_80_Mn_5_Co_5_Fe_10_ precursor primarily removes Al, forming a flake-like structure that significantly increases the surface area. Subsequent secondary dealloying removes Mn, resulting in abundant nanosheets on the material surface. Collectively, these two dealloying steps create a gradient hierarchical pore architecture. This structure facilitates efficient mass transport pathways, which are beneficial for the OER. In contrast, the SEM images of np-CFO (Al) and np-CFO (Mn) (Figure S2) reveal structures consisting solely of bulk plates and nanosheets. Compared to the hierarchical porous nanostructure, these morphologies offer fewer active sites. As shown in Figure 1d, the Co, Fe, O, Al, and Mn elements are uniformly distributed across the catalyst surface. Nevertheless, even after prolonged dealloying, low-intensity detectable signals of Al and Mn indicate trace retention of these elements. These residual Al and Mn species are incorporated as in situ dopants within the host matrix. Interestingly, TEM imaging (Figure 1e) reveals the presence of micro-pores formed during the dealloying process. These loose, porous structures facilitate efficient mass transport, including electrolyte infiltration and oxygen release, thereby synergistically enhancing OER efficiency. Furthermore, as shown in Figure 1f, clearly resolved lattice fringes exhibit a measured spacing of 2.60 Å. This value is slightly larger than the 2.53 Å spacing assigned to the (113) planes of CoFe_2_O_4_. The observed lattice expansion confirms the successful doping of residual Al and Mn, which is consistent with the XRD results presented in Figure 1a.

**Figure 2:**
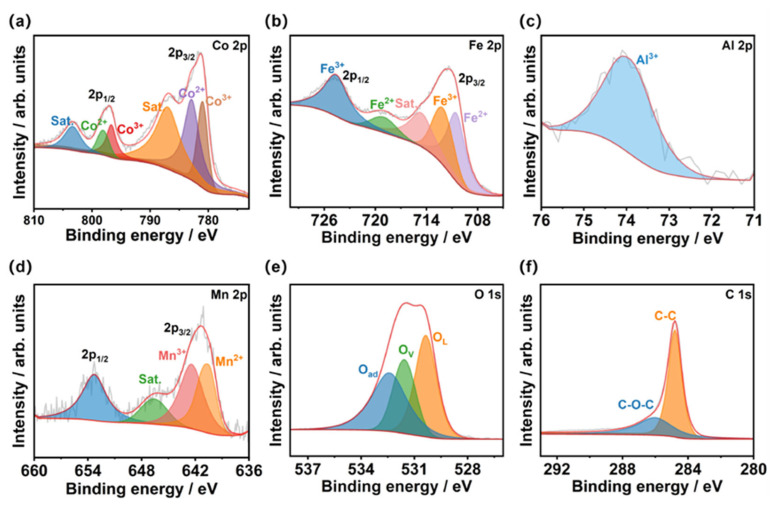
XPS spectra of np-CFO (Al and Mn) electrode: (**a**) Co 2p; (**b**) Fe 2p; (**c**) Al 2p; (**d**) Mn 2p; (**e**) O 1s; and (**f**) C 1s.

XPS analysis is systematically performed to probe the surface chemical state and electronic configuration of the np-CFO (Al and Mn) catalyst. In the high-resolution Co 2p spectrum (Figure 2a), the peaks at approximately 798.12 eV and 782.93 eV correspond to Co^2+^ [21], while those at 796.75 eV and 781.03 eV are attributed to higher-valence Co^3+^ [22]. This indicates that cobalt in the sample primarily exists as Co^2+^ and Co^3+^. In the Fe 2p region (Figure 2b), peaks at 719.32 eV and 710.64 eV correspond to Fe^2+^, while those at 724.76 eV and 712.29 eV are attributed to Fe^3+^ [23,24,25]. This dual-valence state arises from the intrinsic thermodynamic instability of alloyed materials, which spontaneously form surface oxides or hydroxides under ambient conditions [24]. The Al 2p spectrum (Figure 2c) exhibits a peak at 74.05 eV, corresponding to Al^3+^. This confirms the unavoidable presence of residual Al, which likely substitutes Fe^3+^ sites in the structure [26]. In the Mn 2p spectrum (Figure 2d), peaks at 642.47 eV and 640.70 eV correspond to Mn^3+^ and Mn^2+^, respectively, revealing a mixed-valence behavior analogous to the Al case [27,28]. In the O 1s spectrum (Figure 2e), deconvolution reveals three components at 532.43 eV, 531.57 eV, and 530.37 eV, assigned to adsorbed oxygen (O_ad_), oxygen vacancies (O_V_), and lattice oxygen (O_L_) [26]. The presence of these C 1s features indicates surface carbon contamination and oxidation resulting from prolonged air exposure (Figure 2f).

### 3.2. Electrocatalytic OER Activity Analysis

**Figure 3:**
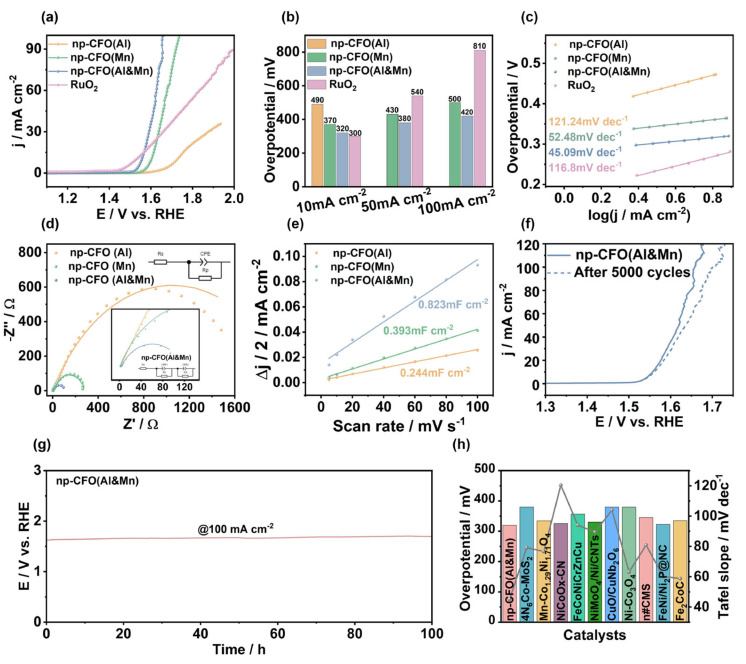
(**a**) LSV curves in 1 M KOH; (**b**) comparison of η_10_, η_50_, and η_100_; (**c**) Tafel plots; (**d**) Nyquist plots, with an inset showing a magnified view of the impedance curve and the fitted equivalent circuit for np-CFO (Al and Mn); (**e**) C_dl_ values; (**f**) LSV curves before and after ADT; (**g**) CP profile at 100 mA cm^−2^; (**h**) performance contrast in 1 M KOH.

The electrocatalytic OER activity of the prepared electrodes is evaluated in 1 M KOH solution. RuO_2_ is selected as the benchmark catalyst for comparison due to its status as a commercially available OER catalyst with established representativeness in water electrolysis catalysis. In the 1 M KOH electrolyte, the np-CFO (Al and Mn) electrode demonstrates a significantly reduced overpotential of 320 mV at 10 mA cm^−2^ (Figure 3a). This value is markedly lower than those of np-CFO (Al) (η_10_ = 490 mV) and np-CFO (Mn) (η_10_ = 370 mV), while slightly exceeding the noble metal benchmark RuO_2_ (η_10_ = 300 mV). Notably, at higher current densities (50 mA cm^−2^ and 100 mA cm^−2^), np-CFO (Al and Mn) exhibits superior performance compared to np-CFO (Al), np-CFO (Mn), and RuO_2_ (Figure 3b). This enhanced electrocatalytic activity is primarily attributed to the dealloying strategy, which optimizes mass transport channels and provides increased active sites. Ex situ XRD analysis after OER testing shows negligible phase change in np-CFO (Al and Mn), revealing its relatively stable phase composition (Figure S3). The Tafel slope serves as a critical kinetic parameter for evaluating electrocatalytic performance, where lower values indicate accelerated OER kinetics due to optimized reaction pathways [29]. As shown in Figure 3c, the Tafel slopes for np-CFO (Al), np-CFO (Mn), np-CFO (Al and Mn), and noble metal RuO_2_ catalysts are measured at 121.24, 52.48, 45.09, and 116.80 mV dec^−1^, respectively. Notably, np-CFO (Al and Mn) exhibits the lowest Tafel slope among all tested materials.

According to the Nyquist plot in Figure 3d and the corresponding fitting parameters in Table S1, np-CFO (Al and Mn) exhibits the lowest charge transfer resistance (Rct) of 113.5 Ω, indicating the highest charge transfer rate, the best conductivity, and the most favorable OER kinetics [30]. To elucidate the origin of OER activity, the electrochemical active surface area (ECSA) of catalysts is estimated from double-layer capacitance (C_dl_) measurements in Figure 3e [31]. Cyclic voltammetry scans are performed within a non-faradaic potential window (0.1–0.2 V vs. RHE) at varying scan rates (5–100 mV s^−1^, Figure S4), effectively mitigating interference from faradaic redox processes. The Cdl values for np-CFO (Al), np-CFO (Mn), and np-CFO (Al and Mn) are calculated as 0.244, 0.393, and 0.823 mF cm^−2^, respectively, derived from the linear slope of the current density difference (Δj/2) at 0.15 V versus the scan rate (Equation (4)). The corresponding ECSAs calculated using Equation (4) are 6.1, 9.825, and 20.575 cm^−2^. This markedly higher ECSA for np-CFO (Al and Mn) originates from dual-cation doping, which constructs a graded hierarchical porous architecture through abundant nanosheet formation on the material surface, thereby providing maximal active sites, which account for its exceptional OER performance. Given that np-CFO (Al and Mn) exhibits superior OER performance compared to np-CFO (Al) and np-CFO (Mn), we select this catalyst to assess its operational durability. Therefore, we conduct accelerated degradation testing (ADT) to validate its stability. As shown in Figure 3f, after 5000 continuous cyclic voltammetry (CV) cycles, the η_100_ value for np-CFO (Al and Mn) shows a minimal increase of merely 50 mV. As evidenced by the XRD pattern and SEM image in Figure S5, the ADT sample maintained its CoFe_2_O_4_ phase structure and exhibited no significant morphological changes after testing, further demonstrating the structural stability of np-CFO (Al and Mn). Furthermore, chronopotentiometry (CP) testing is conducted at 100 mA cm^−2^ for 100 h (Figure 3g). The np-CFO (Al and Mn) electrode demonstrates remarkable stability during prolonged operation, exhibiting negligible potential drift throughout the test. This superior durability stems from the robust skeleton structure formed during the dealloying process, which is virtually indestructible. To investigate the robustness of active sites, np-CFO (Al and Mn) is subjected to thiocyanate poisoning tests. As shown in Figure S6, the OER activity declined by 0.15 mA cm^−2^ relative to the unpoisoned sample, demonstrating compromised stability upon SCN^−^ adsorption. Comparative analysis with state-of-the-art catalysts in the 1 M KOH electrolyte confirms that np-CFO (Al and Mn) serves as a highly active and stable electrode, outperforming recently reported systems across key activity metrics (Figure 3h, Table S2).

**Figure 4:**
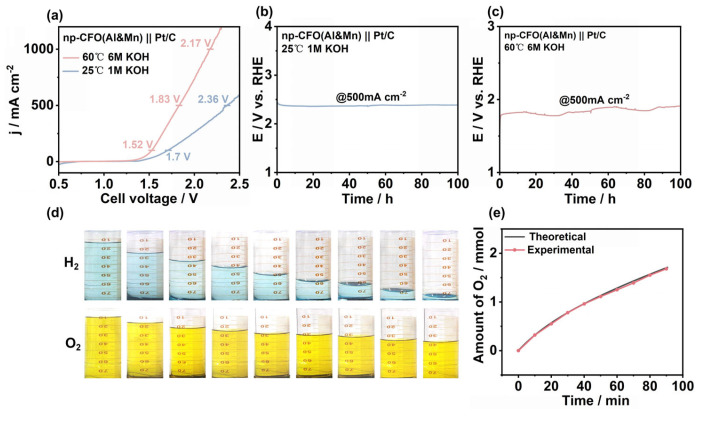
(**a**) LSV curves of np-CFO (Al and Mn) || Pt/C under different conditions; (**b**) CP profiles at 500 mA cm^−2^ in 1M KOH; (**c**) CP profiles at 500 mA cm^−2^ in 6M KOH; (**d**) photographs of H_2_ and O_2_ collected at 2.11 V for 90 min; (**e**) amount of the theoretically calculated and experimentally measured O_2_ production.

Building on the outstanding OER activity of np-CFO (Al and Mn) in 1 M KOH, a symmetrical np-CFO (Al and Mn) || Pt/C structure is constructed to evaluate overall water-splitting performance [32]. As shown in Figure 4a, this system achieves a current density of 100 mA cm^−2^ at just 1.70 V in 1 M KOH (25 °C). Under simulated industrial conditions (6 M KOH, 60 °C), performance is significantly enhanced, requiring remarkably low voltages of 1.52 V, 1.83 V, and 2.17 V to reach 100, 500, and 1000 mA cm^−2^, respectively. Notably, these key metrics surpass recently reported state-of-the-art benchmarks (Table S3). To assess industrial viability, extended durability testing at 500 mA cm^−2^ is performed in both 1 M and 6 M KOH. The system demonstrates robust stability, maintaining continuous operation for 100 h in both electrolytes (Figure 4b,c). Crucially, under harsh 6 M KOH conditions, voltage fluctuations are minimal at 100 mV, highlighting exceptional operational stability for industrial-scale water splitting. Finally, oxygen production is quantified by integrating the simulated AWE. Constant-voltage electrolysis at 2.11 V for 90 min (Figure 4d) yields oxygen evolution data. The experimentally measured oxygen yield shows excellent agreement with the theoretical value (Figure 4e), indicating a Faraday efficiency approaching 100%.

### 3.3. DFT Analysis

**Figure 5:**
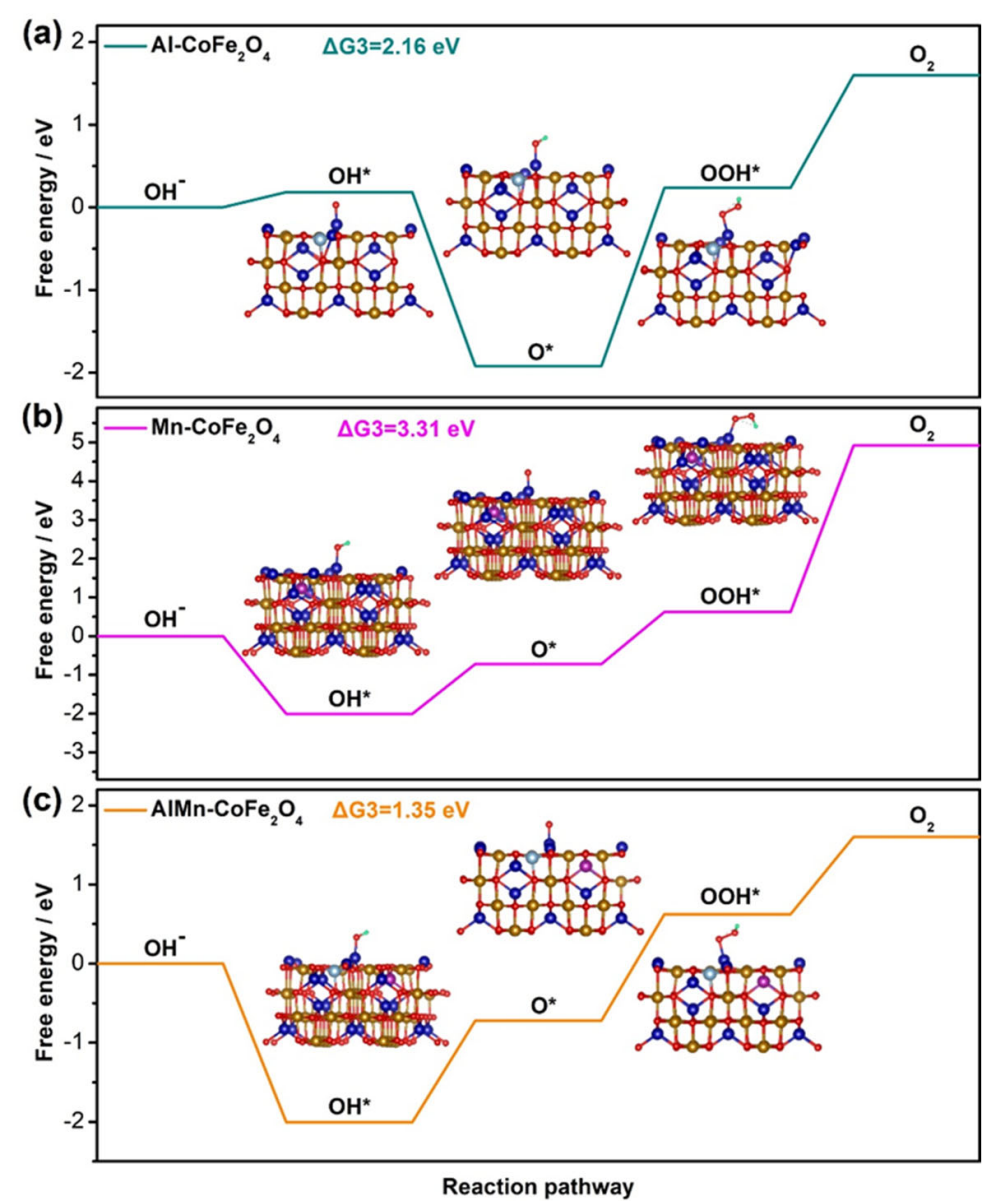
Gibbs free energy diagrams of (**a**) Al-CFO, (**b**) Mn-CFO, and (**c**) AlMn-CFO. Insets are corresponding adsorption models of OER intermediates. Dark blue, brown, light blue, purple, red, and green balls represent Co, Fe, Al, Mn, O, and H atoms, respectively.

Experimental data demonstrate that Al and Mn co-doped CFO delivers enhanced alkaline OER activity relative to its exclusively Al- or Mn-doped counterparts. Theoretical validation of these findings is performed through the construction of Al-doped (Figure S7), Mn-doped (Figure S9), and Al and Mn co-doped models (Figure S11) for the identification of optimal doping sites. Based on the Energy Minimization Principle, Al-CFO-3 (Al-doped), Mn-CFO-2 (Mn-doped), and AlMn-CFO-3 (co-doped) serve as adsorption models for key OER intermediates (*OH, O, OOH). Subsequently, we computationally evaluate multiple oxygen adsorption configurations on Al-CFO-3 (Figure S8), Mn-CFO-2 (Figure S10), and AlMn-CFO-3 (Figure S12). Based on Equations (5)–(8), the *O → *OOH transition is uniformly identified as the rate-determining step (RDS) for Al-CFO, Mn-CFO, and Al Mn-CFO systems, with corresponding energy barriers of 2.16 eV, 3.31 eV, and 1.35 eV, respectively (Figure 5). DFT calculations reveal that Al and Mn-co-doped catalysts exhibit reduced Gibbs free energies in alkaline OER pathways. Finally, the electronic density of states (DOS) in Figure S13 proves that AlMn-CFO possesses a higher electron density compared to Al-CFO and Mn-CFO. That is to say, AlMn-CFO has a faster electron/charge transfer rate and a better conductivity, which would be beneficial to fundamentally enhance electrocatalytic performance.

## 4. Conclusions

In summary, a simple two-step dealloying strategy enables the construction of bimetallic (Al, Mn) co-doped nanoporous spinel CoFe_2_O_4_ (np-CFO) with a tunable structure and composition, serving as a high-efficiency oxygen evolution catalyst. The resulting np-CFO (Al and Mn) exhibits a hierarchical flake architecture, a configuration that simultaneously boosts active site density while enhancing mass transport efficiency. Remarkably, the np-CFO (Al and Mn) catalyst demonstrates exceptionally low oxygen evolution overpotentials in 1 M KOH (η_10_ = 320 mV, η_50_ = 380 mV, and η_100_ = 420 mV), significantly outperforming benchmark catalysts, especially at high current densities, while maintaining exceptional durability. Crucially, under industrial conditions (6 M KOH, 60 °C), the catalyst requires only 1.83 V to achieve 500 mA cm^−2^ and maintains this high current density for 100 h with negligible degradation, showcasing outstanding durability. DFT results confirm that Al and Mn co-doping could indeed improve the adsorption of oxygen-containing intermediates on spinel CFO, which facilitates a reduction in the energy barrier for OER. This work demonstrates the efficacy of bimetallic co-doping in enhancing catalytic performance, offering valuable insights for the industrial development of non-precious metal catalysts, thereby providing critical technical support for engineering applications of industrial-scale green hydrogen production.

## Data Availability

The original contributions presented in this study are included in the article/Supplementary Materials. Further inquiries can be directed to the corresponding author.

## Supplementary Materials

The following supporting information can be downloaded at https://www.mdpi.com/article/10.3390/ma18153633/s1, Figure S1: XRD patterns of initial alloys: (a) Al_85_Co_5_Fe_10_, (b) Mn_70_Co_10_Fe_20_, (c) Al_80_Mn_5_Co_5_Fe_10_; Figure S2: SEM images of (a) np-CFO (Al), (b) np-CFO (Mn); Figure S3: Ex-situ XRD analysis of np-CFO(Al and Mn) before and after OER testing; Figure S4: CV curves of (a) np-CFO (Al), (b) np-CFO (Mn) and (c) np-CFO(Al and Mn) electrodes under different scan rates (5–100 mV s^−1^); Figure S5: (a) XRD pattern and (b) SEM image of np-CFO(Al and Mn) after ADT testing; Figure S6: Chronoamperometric curve of SCN^−^-poisoned np-CFO(Al and Mn) recorded at 1.6 V; Figure S7: (a–d) The slab models of Al-CFO with different Al substitution sites. (e–h) The optimized models and corresponding total energies; Figure S8: (a–e) *O adsorption models of Al-CFO on different metal sites. (f–j) Top view of optimized *O adsorption models. (k–o) Side view and corresponding adsorption energies of optimized *O adsorption models; Figure S9: (a–d) The slab models of Mn-CFO with different Mn substitution sites. (e–h) The optimized models and corresponding total energies; Figure S10: (a–e) *O adsorption models of Mn-CFO on different metal sites. (f–j) Top view of optimized *O adsorption models. (k–o) Side view and corresponding adsorption energies of optimized *O adsorption models; Figure S11: (a–c) The slab models of AlMn-CFO with different AlMn substitution sites. (d–f) The optimized models and corresponding total energies; Figure S12: (a–f) *O adsorption models of AlMn-CFO on different metal sites. (g–l) Top view of optimized *O adsorption models. (m–r) Side view and corresponding adsorption energies of optimized *O adsorption models; Figure S13: DOS of (a) Al-CFO, (b) Mn-CFO and (c) AlMn-CFO; Table S1: Electrochemical impedance parameters obtained by fitting the Nyquist plots of np-CFO(Al), np-CFO(Mn) and np-CFO(Al and Mn) to the equivalent circuit mode; Table S2: Comparison of the electrochemical OER activities of this work with recently reported electrocatalysts under 1 M KOH; Table S3: AWE activities of np-CFO(Al and Mn) || Pt/C couple along with other reported catalysts in 1 M KOH. Refs. [33,34,35,36,37,38,39,40,41,42,43,44] are included in Supplementary Material.
