# Supplementary material for: Hierarchical Flaky Spinel Structure with Al and Mn Co-Doping Towards Preferable Oxygen Evolution Performance
Source: Materials (Basel). 2025 Aug 1;18(15):3633. doi: 10.3390/ma18153633 (PMC12348916; doi:10.3390/ma18153633)
Supplement: Supplementary file 1 [file materials-18-03633-s001.zip › materials-3751844-supplementary.pdf]

Hierarchical Flaky Spinel Structure with Al and Mn Co-Doping Towards Preferable Oxygen Evolution Performance

Hengfen Shen [1], Hao Du [2], Peng Li [2] and Mei Wang [1,*]

[1] School of Materials Science and Engineering, North University of China, Taiyuan 030051, China
[2] School of Energy and Power Engineering, North University of China, Taiyuan 030051, China
* Correspondence: wangmei@nuc.edu.cn

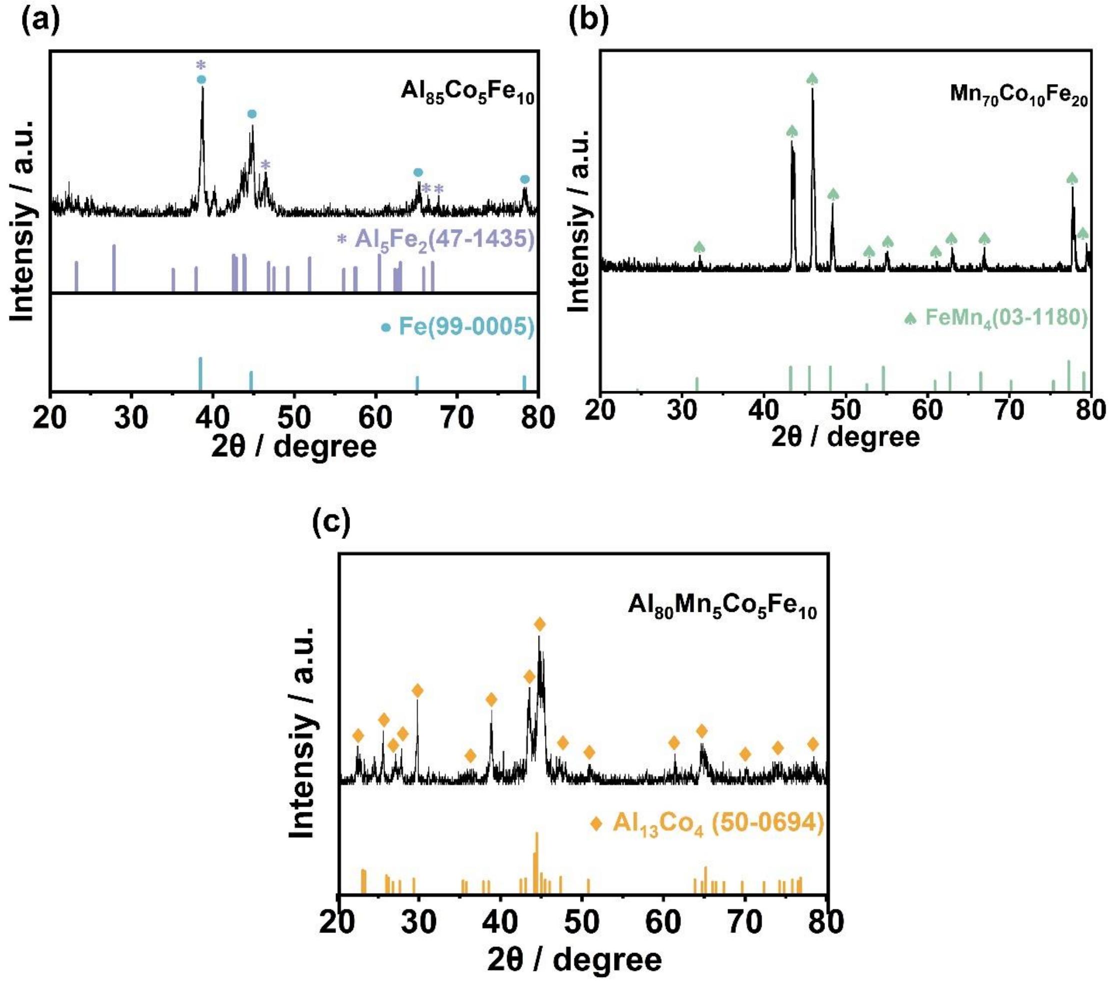

Figure S1. XRD patterns of initial alloys: (a) $Al_{85}Co_5Fe_{10}$, (b) $Mn_{70}Co_{10}Fe_{20}$, (c) $Al_{80}Mn_5Co_5Fe_{10}$.

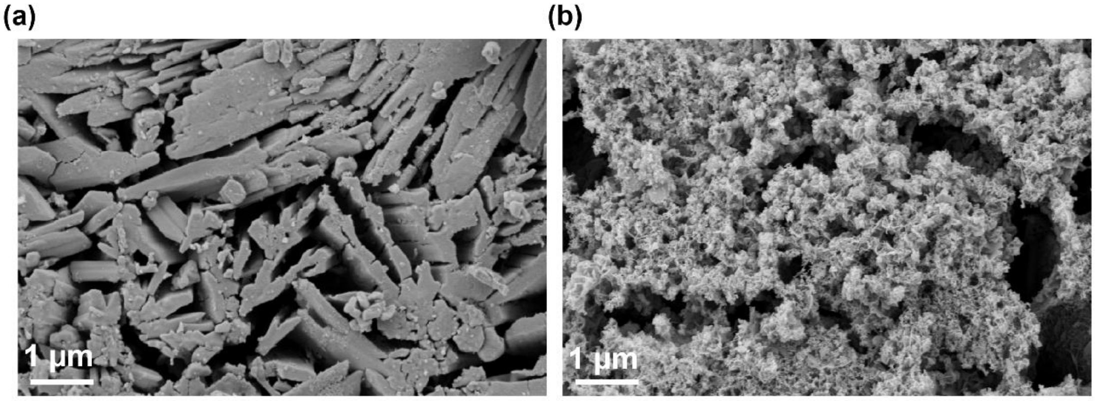

Figure S2. SEM images of (a) np-CFO (Al), (b) np-CFO (Mn).

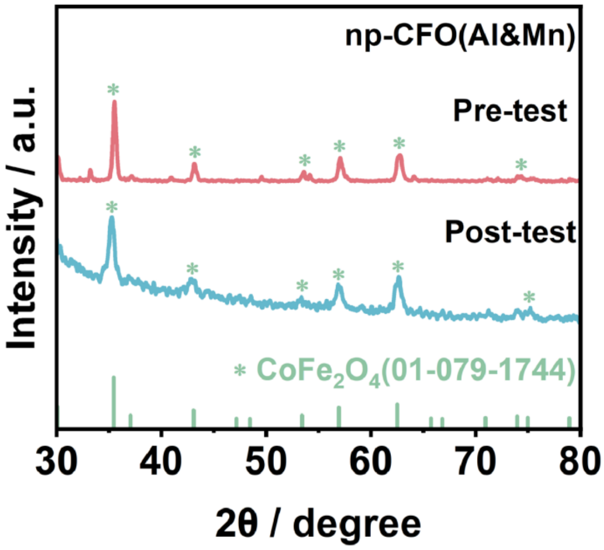

Figure S3. Ex-situ XRD analysis of np-CFO(Al&Mn) before and after OER testing.

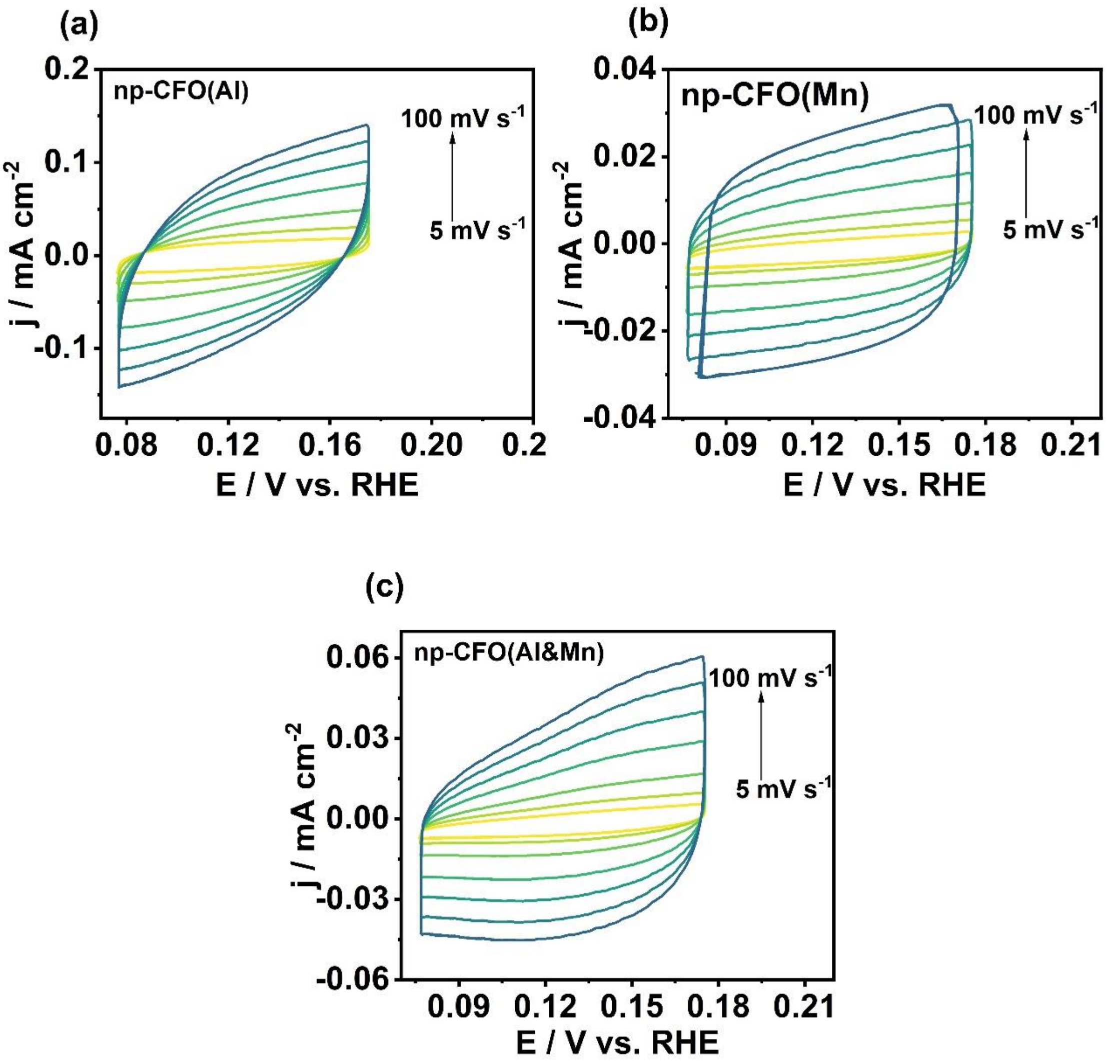

Figure S4. CV curves of (a) np-CFO (Al), (b) np-CFO (Mn) and (c) np-CFO(Al&Mn) electrodes under different scan rates (5-100 mV s^{-1}).

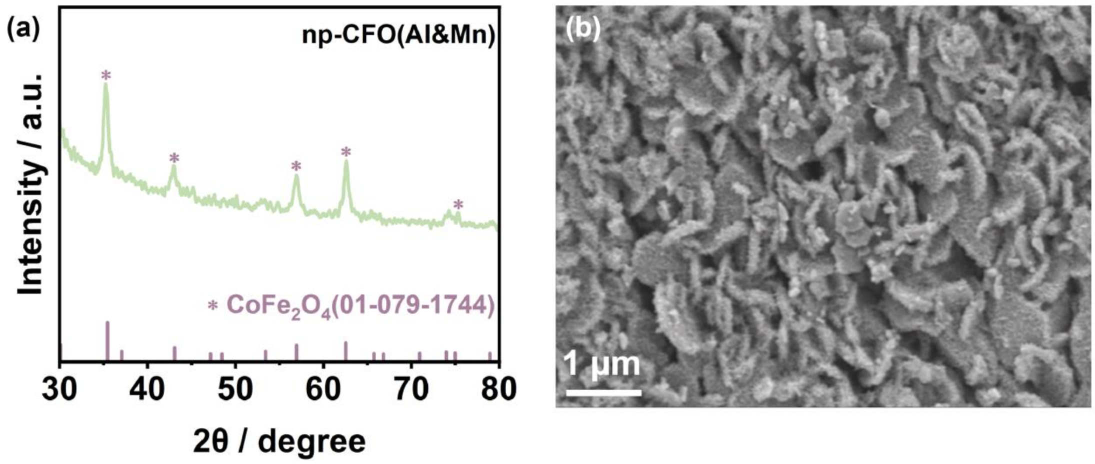

Figure S5. (a) XRD pattern and (b) SEM image of np-CFO(Al&Mn) after ADT testing.

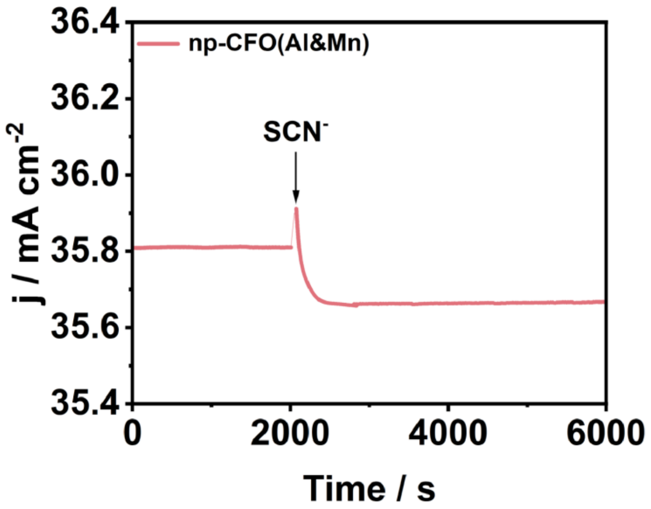

Figure S6. Chronoamperometric curve of SCN^--poisoned np-CFO(Al&Mn) recorded at 1.6 V.

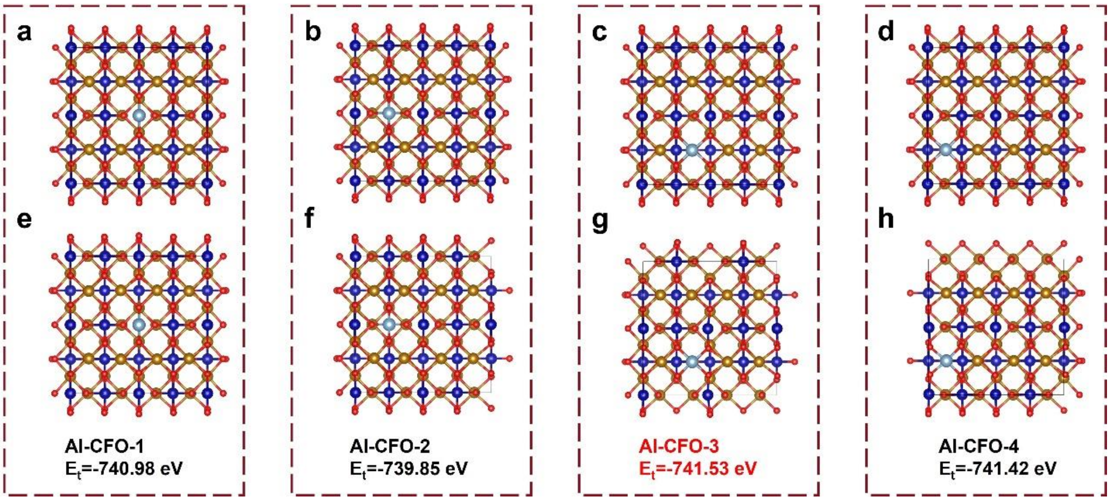

Figure S7. (a-d) The slab models of Al-CFO with different Al substitution sites. (e-h) The optimized models and corresponding total energies.

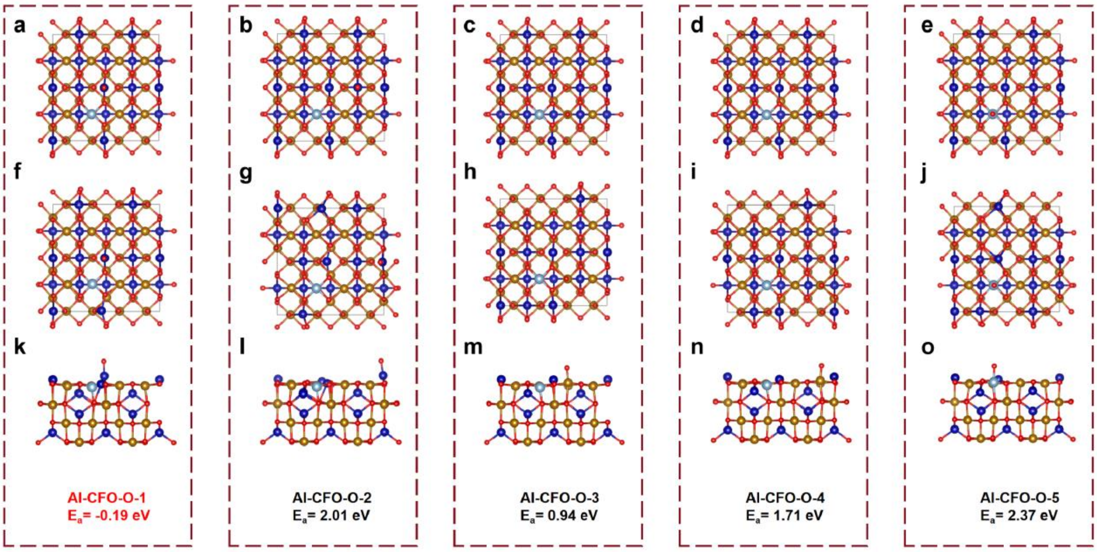

Figure S8. (a-e) *O adsorption models of Al-CFO on different metal sites. (f-j) Top view of optimized *O adsorption models. (k-o) Side view and corresponding adsorption energies of optimized *O adsorption models.

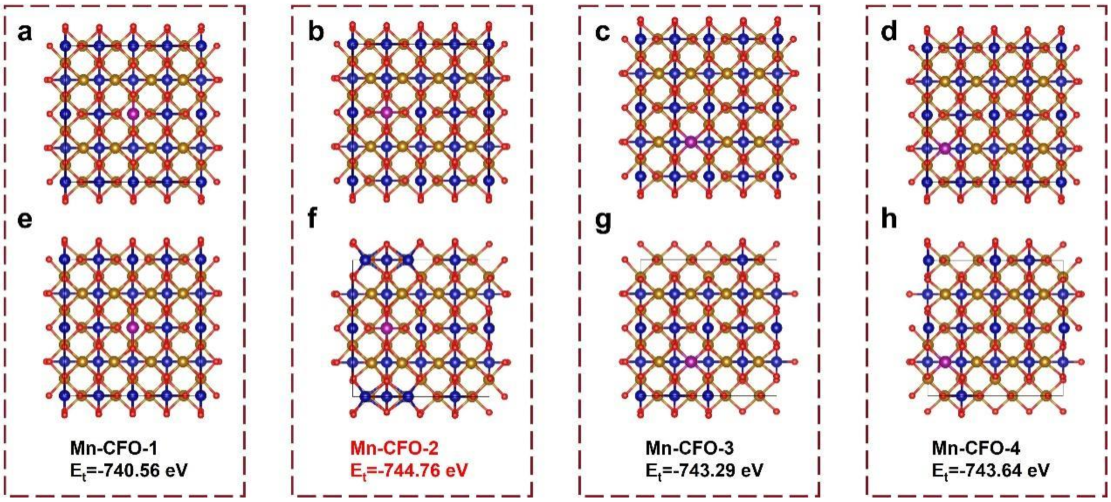

Figure S9. (a-d) The slab models of Mn-CFO with different Mn substitution sites. (e-h) The optimized models and corresponding total energies.

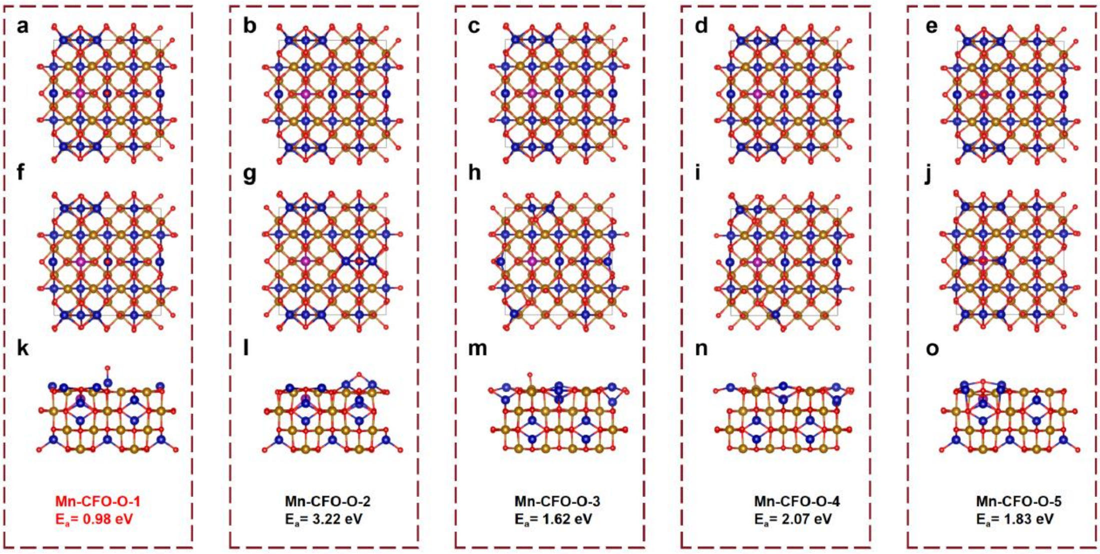

Figure S10. (a-e) *O adsorption models of Mn-CFO on different metal sites. (f-j) Top view of optimized *O adsorption models. (k-o) Side view and corresponding adsorption energies of optimized *O adsorption models.

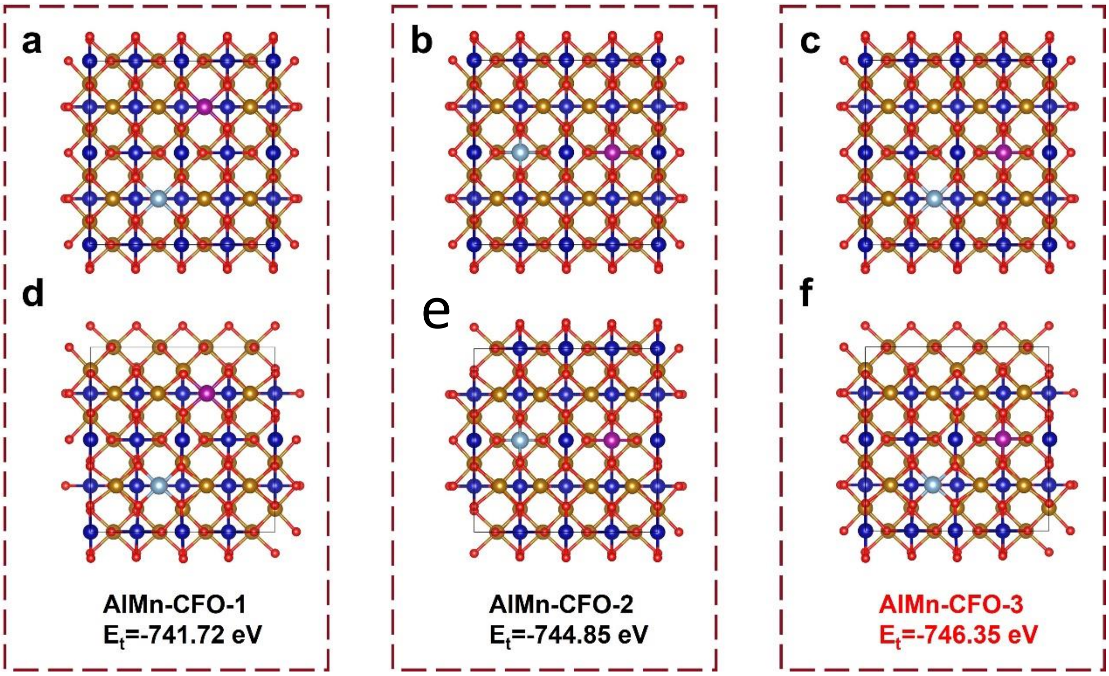

Figure S11. (a-c) The slab models of AlMn-CFO with different AlMn substitution sites. (d-f) The optimized models and corresponding total energies.

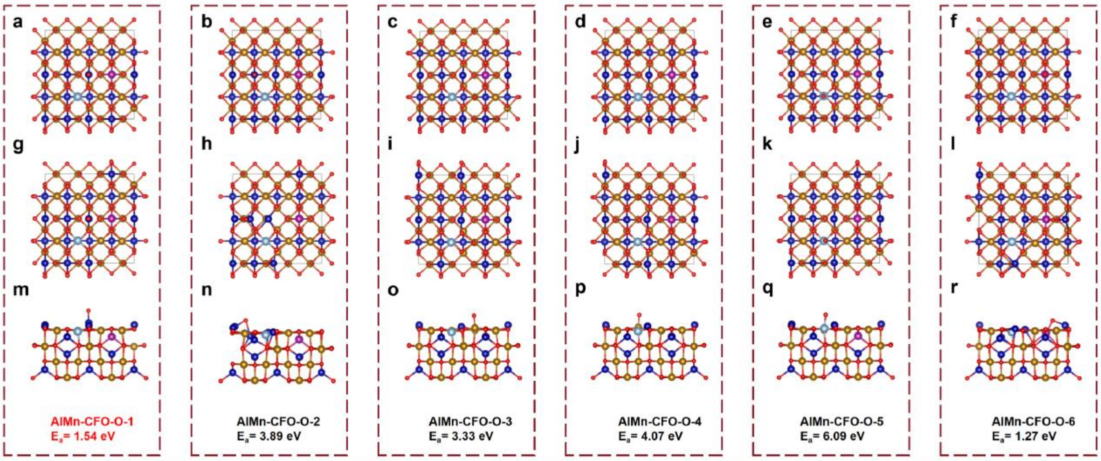

Figure S12. (a-f) *O adsorption models of AlMn-CFO on different metal sites. (g-l) Top view of optimized *O adsorption models. (m-r) Side view and corresponding adsorption energies of optimized *O adsorption models.

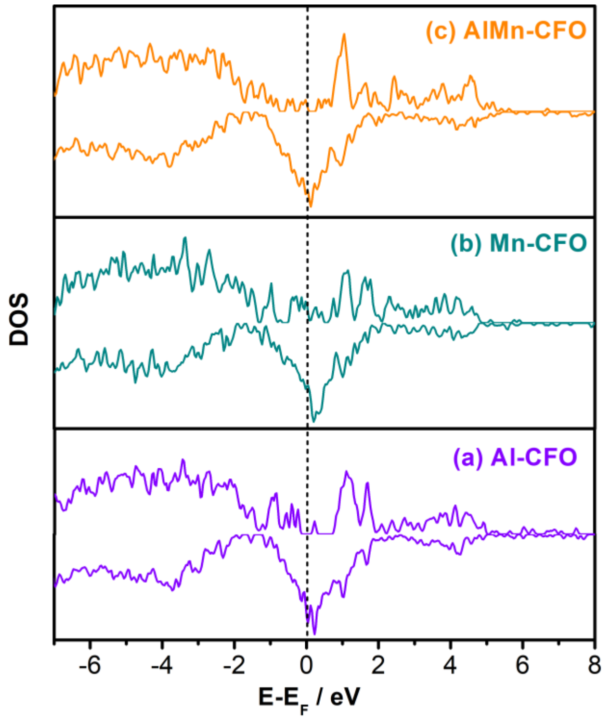

Figure S13. DOS of (a) Al-CFO, (b) Mn-CFO and (c) AlMn-CFO.

Table S1. Electrochemical impedance parameters obtained by fitting the Nyquist plots of np-CFO(Al), np-CFO(Mn) and np-CFO(Al&Mn) to the equivalent circuit mode.

Catalysts	Rs	CPE1-T	CPE1-P	R0	CPE2-T	CPE2-P	Rct
np-CFO (Al)	1.6084	1.6702E-2	0.67032	0	0	0	2101.6
np-CFO (Mn)	1.4084	2.50122E-3	0.2701	0	0	0	284.66
**np-CFO (Al&Mn)**	**2.9089**	**1.3053E-3**	**0.8228**	**1.5367**	**2.1223E-2**	**0.66358**	**113.5**

Table S2. Comparison of the electrochemical OER activities of this work with recently reported electrocatalysts under 1 M KOH.

	η_{10} (mV)	Tafel slope (mV dec^{-1})	Reference
**np-CFO(Al&Mn)**	**320**	**45.09**	**This work**
FeNi/Ni_2P@NC	323	60.3	[1]
NiCoOx-CN	326	120.4	[2]
$NiMoO_4$/Ni/CNTs	330	89.5	[3]
Mn-$Co_{1.29}Ni_{1.71}O_4$	334.3	76.7	[4]
Fe_2Co/C	335	58.8	[5]
n#CMS	345	80.89	[6]
FeCoNiCrZnCu	357	94	[7]
Ni-Co_3O_4	380	63.19	[8]
$4N_6Co$-MoS_2	380	79.2	[9]
CuO/$CuNb_2O_6$	380	104	[10]

Table S3. AWE activities of np-CFO(Al&Mn) || Pt/C couple along with other reported catalysts in 1 M KOH.

Couple	Cell voltage (V) @100 mA cm^{-2}	Condition	Reference
**np-CFO (Al&Mn)‖ Pt/C**	**1.52V**	**25°C 1MKOH**	**This work**
**$NiCu/NiMoO_4/NF$‖$NiCu/NiMoO_4/NF$**	1.72	25°C 1MKOH	[11]
**NiFe/NF ‖NiFe/NF**	1.75	25°C 1MKOH	[12]
**$CoCrFeNiMoAl_{0.6}$‖$CoCrFeNiMoAl_{0.6}$**	1.83	25°C 1MKOH	[13]
**$NiSe_2/CoSe/NF$-3.0‖$NiSe_2/CoSe/NF$-3.0**	1.85	25°C 1MKOH	[14]
**$4N_6Co-MoS_2$‖ $4N_6Co-MoS_2$**	2.2	25°C 1MKOH	[9]

REFERENCES

1. Yu, T. R.; Zhang, Y. H.; Zhou, J. Q.; Feng, M. X.; Zhang, Z. W.; Zhou, Y. M. FeNi/Ni_2P nanoparticles encapsulated in nitrogen-doped porous carbon: efficient electrocatalysts for oxygen evolution reaction. *JOURNAL OF MATERIALS SCIENCE*. **2024**, *59* (47), 21710-21720.
2. Wang, X.; Dai, Y.; Wang, P.; Yue, D. Melamine intercalation approach for the synthesis of C, N codoped net-like NiCo oxides composites and Its Application towards oxygen evolution reaction in alkaline solution. *International Journal of Hydrogen Energy*. **2024**, *51*, 1407-1416.
3. Li, G.-L.; Qiao, X.-Y.; Miao, Y.-Y.; Wang, T.-Y.; Deng, F. Synergistic Effect of N-$NiMoO_4$/Ni Heterogeneous Interface with Oxygen Vacancies in N-$NiMoO_4$/Ni/CNTs for Superior Overall Water Splitting. *Small*. **2023**, *19* (28), 2207196.
4. Cheng, Y. J.; Guo, X. Y.; Ma, Z. Z.; Dong, K. H.; Miao, L. H.; Du, S. Highly Efficient and Stable Mn-$Co_{1.29}Ni_{1.71}O_4$ Electrocatalysts for Alkaline Water Electrolysis: Atomic Doping Strategy for Enhanced OER and HER Performance. *MOLECULES*. **2025**, *30* (5), 1165.
5. Guo, J.; Gao, Y.; Cao, X.; Rong, X.; Chi, S.; Fan, G.; Zhang, L.; Tian, G.; Zhao, X. Fe-Co bimetallic MOF-derived carbon directly application as an efficient electrocatalyst for oxygen evolution reaction. *Inorganic Chemistry Communications*. **2024**, *170* (9), 113394.
6. Zeng, T.; Lin, J.; Wang, B.; Guo, B.; Ding, Y.; Sun, C. Q. Reducing the overpotential of overall water spitting by micro-pump-like electrode engineering. *Chemical Engineering Journal*. **2024**, *500*, 156788.
7. Li, M.; Ye, X.; Guo, S.; Hou, M.; Yang, L.; Chen, K.; Gao, L.; Li, Y.; Briois, P. High entropy design endows spinel compounds with excellent bifunctional catalysis for hydrogen production from water electrolysis. *International Journal of Hydrogen Energy*. **2025**, *130* (3), 644-653.
8. Vazhayil, A.; Ashok C, S.; Thomas, N. Probing the electrocatalytic activity of hierarchically mesoporous M-Co_3O_4 (M = Ni, Zn, and Mn) with branched pattern for oxygen evolution reaction. *Journal of Electroanalytical Chemistry*. **2023**, *934* (10), 117298.
9. Lu, Y.-Z.; Wei, S.-Z.; Yang, S.-S.; Fu, L.-P.; Tang, J.-Q.; Liu, Y.; Liu, W. Hydrothermal synthesis and bifunctional electrocatalytic properties of N and Co co-doped MoS_2 for water splitting. *Tungsten*. **2025**.
10. Alves, H. P. A.; Pereira, T. O.; Raimundo, R. A.; Alves, R. F.; Junior, R. A.; Campos, L. F. A.; Macedo, D. A.; Medeiros, E. S. Cu–Nb–O ternary system nanofibers obtained by solution blow spinning as catalysts for oxygen evolution reaction. *Journal of Physics and Chemistry of Solids*. **2025**, *199*, 112525.
11. Huang, B.; Wei, D.; Meng, Z.; Liu, Q.; Ren, Y.; Deng, Z.; Shi, C.; Xu, M. Regulating oxygen vacancy and interface structure of NiCu/$NiMoO_4$ bifunctional catalyst for stable and efficient photothermal catalysis and overall water splitting. *Journal of Alloys and Compounds*. **2025**, *1028*, 180701.
12. Sun, Y.; Yang, F.; Sun, S.; Wei, K.; Wang, Y.; Ma, G.; An, J.; Yuan, J.; Zhao, M.; Liu, J.; Liu, H.; Li, Y. Phase regulation of $Ni(OH)_2$ nanosheets induced by W doping as self-supporting electrodes for boosted water electrolysis. *Journal of Colloid and Interface Science*. **2025**, *684*, 1-10.
13. Huo, X.; Zhang, Y.; Zhou, G.; Zhong, J.; Zuo, X.; Zhang, N.; Xu, X. Plasma-sprayed porous high-entropy alloy coatings as cost-effective and durable catalysts for efficient alkaline water splitting. *Journal of Alloys and Compounds*. **2025**, *1026*, 180461.

14. Zhao, X.; Shang, Z.; Li, N.; Lu, Q.; Guo, E.; Wei, M.; Ji, X.-Y.; Yang, Z.; Liu, X. NH4F-induced morphology-dependent $NiSe_2$/CoSe electrocatalysts on Ni foam for enhanced overall water splitting. *Journal of Colloid and Interface Science*. **2025**, *693*, 137642.
